# β-N-Methylamino-L-alanine Induces Neurological Deficits and Shortened Life Span in *Drosophila*

**DOI:** 10.3390/toxins2112663

**Published:** 2010-11-03

**Authors:** Xianchong Zhou, Wilfredo Escala, Spyridon Papapetropoulos, R. Grace Zhai

**Affiliations:** 1 Department of Molecular and Cellular Pharmacology, Miller School of Medicine, University of Miami, Miami, Florida 33136, USA; Email: zhouxchong@gmail.com; 2 University of Miami Bridges to the Future Program, Miami Dade Honors College, Miami, Florida 33132, USA; Email: wilfredo.escala@gmail.com; 3 Department of Neurology, Miller School of Medicine, University of Miami, Miami, Florida 33136, USA; Email: spapapetropoulos@med.miami.edu

**Keywords:** Amyotrophic Lateral Sclerosis, dementia, neurodegeneration

## Abstract

The neurotoxic non-protein amino acid, β-N-methylamino-L-alanine (BMAA), was first associated with the high incidence of Amyotrophic Lateral Sclerosis/Parkinsonism Dementia Complex (ALS/PDC) in Guam. Recently, BMAA has been implicated as a fierce environmental factor that contributes to the etiology of Alzheimer’s and Parkinson’s diseases, in addition to ALS. However, the toxicity of BMAA *in vivo* has not been clearly demonstrated. Here we report our investigation of the neurotoxicity of BMAA in *Drosophila*. We found that dietary intake of BMAA reduced life span, locomotor functions, and learning and memory abilities in flies. The severity of the alterations in phenotype is correlated with the concentration of BMAA detected in flies. Interestingly, developmental exposure to BMAA had limited impact on survival rate, but reduced fertility in females, and caused delayed neurological impairment in aged adults. Our studies indicate that BMAA exposure causes chronic neurotoxicity, and that *Drosophila* serves as a useful model in dissecting the pathogenesis of ALS/PDC.

## 1. Introduction

Alzheimer’s and Parkinson’s diseases and Amyotrophic Lateral Sclerosis (ALS) are the most common age-related neurodegenerative diseases worldwide. Most cases (90–95%) of these diseases are sporadic and have essentially no known genetic component. Previous cytotoxic studies flourished the awareness of high ALS incidence on the South Pacific island of Guam and in conjunction with dietary epidemiological observations, these studies have allowed for the isolation of a potential neurotoxin, β-N-methylamino-l-alanine (BMAA) [1,2]. BMAA is a neurotoxic, non-lipophilic, non-protein amino acid produced by cyanobacteria. In the Guam ecosystem BMAA is produced by cyanobacteria of the genus *Nostoc*, which are root symbionts of cycads, and is then biomagnified in cycad seeds and in Marianas flying foxes (*Pteropus mariannus*) that forage on them [3]. Consumption of flying foxes by the Chamorro people has been implicated as a delivery mechanism for relatively high doses of BMAA in the Chamorro diet [1]. BMAA occurs not only as a free amino acid in the ecosystem but also can be released from a protein-bound form by acid hydrolysis [4]. BMAA has been detected in both free and protein-bound form in the brain tissues of Chamorro patients who died from ALS/PDC [4].

Significantly, BMAA is not restricted to Guam but is rather broadly present in the environment. High concentrations of BMAA have been found to be associated with cyanobacterial blooms outside of Guam, in marine [5] and fresh water [6] environments. Recently, the occurrence and bioaccumulation of BMAA was detected in a temperate aquatic ecosystem (Baltic Sea), suggesting a potential widespread human exposure [7]. It is interesting to note that different forms of cyanobacteria are used as dietary items in several indigenous cultures around the world, and recently BMAA was detected in *llullucha*, which are colonies of cyanobacteria consumed by Peruvian highlanders [8]. The health effects of these foods remain to be examined. Evidence supporting the global presence of BMAA and the biomagnification of cyanobacterial BMAA came from recent studies that reported detection of BMAA in brain tissue from ALS, Parkinson’s disease (PD) and Alzheimer’s disease (AD) patients in the United States and Canada, whereas no or very low levels of BMAA were detected in brain tissue from subjects without clinical or pathological evidence of ALS or AD and in other ALS patient series [9,10,11,12,13]. The significant concentration of BMAA in selected human disease patients outside Guam suggests the existence of alternative ecological pathways for the bioaccumulation of BMAA in aquatic or terrestrial ecosystems in continental America, and more importantly a possible role of BMAA in the pathogenesis of multiple neurodegenerative diseases. These observations imply that BMAA could be one of the first environmental factors that contribute to the etiology of AD, PD and ALS.

BMAA is a non-protein amino acid that structurally appears as a methylated alanine. Several studies using cultured neurons have indicated that BMAA induced neuronal cell death [14] and preferentially injured spinal motor neurons *in vitro* [15]. Inasmuch as the carbamate modification of BMAA under physiological conditions in the presence of bicarbonate structurally resembles glutamate [16], it has been postulated that BMAA may act as an agonist for AMPA/kainite receptors [15], NMDA and mGluR5 receptors [14], or may mediate neurodegeneration via an excitotoxic mechanism induced by the activation of excitatory amino acid (EAA) receptors [17]. However, most of these studies have been done on cultured brain cells, and the toxicity of BMAA *in vivo* has not been demonstrated clearly and remains a controversial issue [18]. Furthermore, it is unknown what concentration of BMAA is toxic or detrimental and whether the concentration of BMAA in the brain is correlated with neurodegeneration. The BMAA toxicology field has reached the stage where the development of animal models is crucial [19]. To address these questions we used a recently established BMAA feeding paradigm in fruit fly *Drosophila* *melanogaster* [20] to examine the toxicity of BMAA on survival and neurological functions. Here, we report our findings that dietary intake of BMAA reduced the life span as well as the locomotor abilities and learning and memory functions of *Drosophila*. We have also found that the severity of the alterations in phenotype was proportional to the concentration of BMAA in the feeding medium and was correlated with the presence of free BMAA in the fly brain. Furthermore, the findings in this work show that BMAA feeding during development had a limited impact on the survival rate or the progress of development; but resulted in reduced fertility in females and caused delayed neurological impairment in aged adults. Together, these results provide the first experimental evidence for progressive neurotoxicity of BMAA. In summary, the *Drosophila* model allowed us to evaluate the neurotoxicity of BMAA *in vivo*, and our results here provide a link between the neurological deficits and the concentrations of free BMAA in the nervous system.

## 2. Materials and Methods

### 2.1. *Drosophila* Culture and Drug Feeding

Wild-type *Canton S* (*CS*) flies were raised on corn meal/yeast/molasses media at room temperature (20 °C) under ambient light. For BMAA and other amino acid feeding experiments, BMAA, Glutamic acid or Alanine (B107, G1251 or A7627, Sigma-Aldrich, St. Louis, U.S.) were dissolved in water and mixed into sucrose/agar medium. The final concentration of the medium was 5% sucrose, 1% agar, and 4 mM of amino acid. *CS* male and female flies 1–3 days in age were separated, and twenty flies were put into each vial containing 2 mL sucrose/agar medium. The numbers of dead and live flies were recorded daily. Dead flies were distinguished by counting the number of flies that remained inanimate for over 15 seconds after a shaking of the vial. About 200-300 flies were used for each experimental group. The amino acid-containing medium was replaced once a week.

### 2.2. Negative Geotaxic Assay

The negative geotaxis assay (climbing assay) was performed as described [21,22]. Flies were sexed and placed in empty polystyrene vials in groups of ten and allowed 10 minutes to acclimatize. In the behavior assay, flies were gently tapped to the bottom of the vial and then allowed to crawl. In response to tapping (gentle agitation), flies crawl/climb upward against gravity. The number of flies passing 8 cm within 10 seconds was counted. Ten trials were performed for each group. Ten to twenty groups (100 to 200 flies) were tested for each experimental condition. Only viable flies were used in climbing assays, and after each assay, the viability of flies was retested. Any flies that died during the course of the assay were eliminated and the data points associated with dead flies were disregarded.

### 2.3. Aversive Phototaxic Suppression Assay

The aversive phototaxic suppression assay measures the learning and memory ability of flies. It was carried out as described [23,24] with a modified T-maze where the maze chamber was connected with two horizontal vials—a darkened vial externally covered by foil paper and a lighted vial connected to a light source. Filter paper soaked with 400 μL of a 1 µM quinine hydrochloride solution was placed in the lighted vial. Wild-type (Canton S) female flies 1–3 days old were fed with control 1% sugar/5% sucrose media or 1% sugar/5% sucrose media with 4mM L-BMAA, glutamate or alanine for three days. On day four, these female flies were subjected to the aversive phototaxic suppression assay. The assay was divided into two phases, a conditioning phase and a test phase. For conditioning, a fly was placed in the darkened vial and given a 30 second acclimatization period. Then the trap door that divides both vials was opened so that the fly could cross toward the lighted vial. The fly was then given 25 seconds to cross to the lighted vial. Any fly that showed any locomotor or phototactic defects and did not cross over to the lighted vial within 25 seconds in the first conditioning trial was discarded. At the end of this (25 second) time period, the light was turned off, and the fly was quickly and gently tapped back into the darkened vial followed by closing the trap door. After 15 seconds of acclimatization in the darkened vial, the light was turned on and the trap door was opened. The fly was given 25 seconds to cross over to the lighted vial. This process was repeated ten times to complete the conditioning phase. Five test trials were performed immediately post-conditioning (testing learning abilities), and six hours post-conditioning (testing memory abilities). A pass response was one where the fly refused to cross toward the lighted vial and a fail response was one where the fly entered into the lighted vial. The number of pass responses was recorded and the success rate was calculated for each fly. Learning and memory index for each treatment group was plotted as the mean of the success rate of the qualified flies that completed the conditioning phase. Forty to fifty flies from each group were collected and subjected to the test and twenty to twenty five flies that completed the conditioning phase were included.

### 2.4. Tissue Sample Preparation for HPLC

The tissue preparation procedure was optimized for fly tissue based on a previously described sample preparation protocol for cyanobacteria extraction [5,20]. Adult flies were anesthetized by carbon dioxide, collected into eppendorf tubes and flash-frozen to −80 °C. For extraction 450 µL of 70% methanol was added and lysis was achieved by three freeze-thaw cycles in liquid nitrogen, followed by three cycles of sonication for 20 seconds (Branson Sonifier 150). Samples were kept on ice to minimize protein degradation. Samples were then dried by centrifugal vacuum concentration at 4 °C. The dry weight of samples was measured and recorded. The dried material was then resuspended in 250 µL of 70% methanol, and 4 volumes (1 mL) of acetone, kept at −20 °C, was added. The proteins were precipitated at −20 °C for a minimum of three hours and centrifuged for 20 minutes at 4 °C at 15000xg. The supernatant containing free amino acids was collected and filtered with an Amicon spin-filter (Millipore, UFC30GV00). The protein pellet containing bound amino acids was hydrolyzed in 500 µL 6M HCl in closed glass vials at 110 °C for 24 hours, filtered with an Amicon spin-filter (Millipore), and dried overnight at 55 °C. The sample was then resuspended in 110 µL of 20 mM HCl.

### 2.5. Detection of BMAA with HPLC Analysis

A fluorescence detection method for BMAA was used as described previously [20]. The detection limit of L-BMAA in fly tissue is 4 ng BMAA/1.6 µg sample [20]. The samples were diluted and derivatized with 6-aminoquinolyl-*N*-hydroxysuccinimidyl carbamate (Waters AQC Tag reagent, WAT052880) at a concentration of 20% AQC tag in 0.5 M borate buffer.

The HPLC system (Waters, U.S.) used in this study consisted of a binary HPLC pump (Waters 1525), an autosampler (Waters 717), and a scanning fluorescence detector (Waters 474). Separation of amino acids was carried out using a Nova-Pak C18 Column (4 µm, 3.9 × 300 mm, Waters, WAT 011695), joined with a Nova-Pak®C18 Guard Column (4 µm, 3.9 × 20 mm, WAT044380). The column was thermostated at 37 °C. The flow rate was set to 1.0 mL/min and the injection volume was 10 µL. The excitation and emission peak wavelengths for fluorescence detection of derivatized BMAA were 250 nm and 395 nm. Mobile phase A consisted of a dilution 1:11 of Eluent A concentrate (WAT052890) set to pH 5.2 prepared with HPLC grade water (VWR 4218-03). Eluent B consisted of 52% HPLC grade acetonitrile (Sigma 34851) and 48% HPLC grade water. Gradient conditions were as described [5]. The HPLC chromatographs were acquired and then analyzed by Empower II software (Waters).

## 3. Results

### 3.1. Dietary Intake of BMAA Reduced Life Span and Induced Locomotor and Learning and Memory Deficits in a Dose-dependent Manner

The fruit fly *Drosophila* has been used to study human neurodegenerative diseases, including ALS, and these studies have contributed greatly to our understanding of the mechanisms of neurodegeneration [25,26,27,28]. Specifically, *Drosophila* has been shown to be an ideal model to quantitatively measure motor coordination and learning and memory, as flies have distinctive behaviors that are stereotypic and quantifiable [29], such as geotaxic and phototaxic behavior [30,31]. Geotaxis refers to the climbing behavior of flies and their natural tendency to climb against gravity, where a reduced climbing behavior indicates reduced motor coordination [21,22]. Phototaxis refers to the instinctive behavior of flies to move toward light [32].

**Figure 1:**
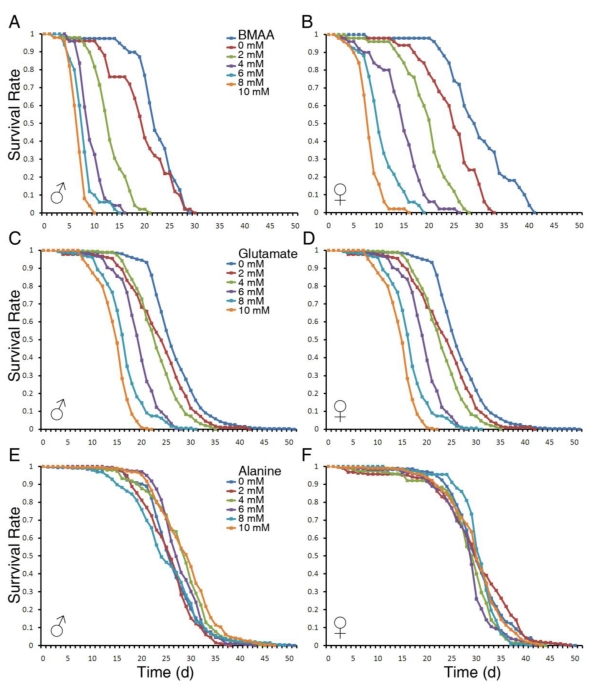
BMAA severely reduced the life span of *Drosophila melanogaster* in a dose‑dependent manner. Adult flies from 1–3 days old were collected, separated as males and females, and raised on a medium consisting of 5% sugar, 1% agar and 0 to 10 mM of BMAA, glutamate or alanine. About 200 flies were tested in each treatment group. The numbers of live and dead flies were recorded daily, and the survival rate was calculated and plotted. Represented panels show the survival curves of male and female flies raised on medium containing BMAA (A, B), glutamate (C, D) and alanine (E, F).

To investigate the toxicity of BMAA on adult flies, we first measured the effects of dietary intake of BMAA on life span. Adult flies of 1–3 days of age were raised on sucrose/agar media containing 0, 2, 4, 6, 8 or 10 mM of BMAA. Because BMAA is structurally similar to alanine in its native form and to glutamate in its carbamate form [16], we included alanine and glutamate as controls. As shown in Figure 1, BMAA severely reduced the life span of both male and female flies in a dose-dependent manner (Figure 1A and 1B). Glutamate also reduced life span, but to a lesser extent (Figure 1C and 1D), and alanine had no effect on the life span of adult flies (Figure 1E and 1F). Overall, males were more susceptible to BMAA as the survival rate of males at each dosage was lower than that of females.

**Figure 2:**
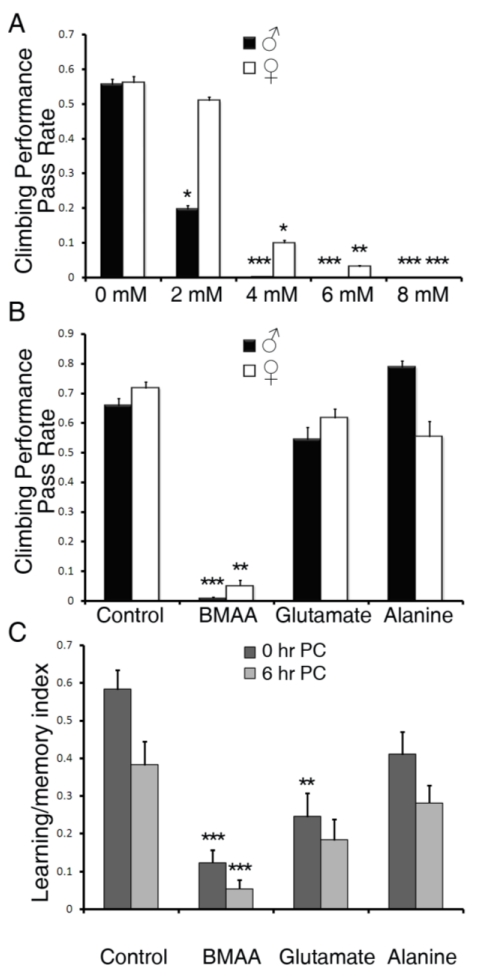
BMAA reduced geotactic behavior and learning and memory abilities. (A-B) Climbing tests were done on adult flies fed on sugar/agar medium containing BMAA, glutamate, or alanine for 7 days. The climbing performance was calculated by the percentage of flies that passed 8 cm within a ten second period. (A) Climbing performance of adult flies fed with 0 to 8 mM BMAA. (B) Climbing performance of adult flies fed with 4 mM BMAA, glutamate or alanine. At least 200 flies were tested for each group. All data were presented as mean ± S.E.M. n = 10. (C) The aversive phototaxic suppression assay was done on adult female flies that fed on sugar/agar medium containing BMAA, glutamate, or alanine for 3 days. The learning and memory index was calculated by the success rate of flies that avoided the lighted chamber containing quinine within 25 seconds during the testing phase of the assay. Forty to 50 flies from each group were collected and subjected to the test and at least 20 flies that completed the conditioning phase were included for each group. All data were presented as mean ± S.E.M. n ≥ 20. Significance level was established by one way ANOVA post-hoc Scheffe test. * p ≤ 0.05, ** p ≤ 0.01, *** p ≤ 0.001.

Next we examined the effects of BMAA on geotaxic behavior. We measured the climbing performance of viable flies at day seven of BMAA feeding, as this time point marked a survival rate of 50% or higher at all concentrations (Figure 1). As shown in Figure 2A, BMAA reduced the climbing performance in a dose-dependent manner. Interestingly, male flies were more susceptible to BMAA, as 2 mM BMAA significantly reduced the climbing performance in male flies, but not in females. No successful climbing behavior was observed in flies fed with 8 or 10 mM of BMAA (Figure 2A and data not shown). Compared to glutamate and alanine at 4 mM, only BMAA affected the geotaxic behavior (Figure 2B), indicating the specific neurotoxicity mediated by BMAA.

In order to test the specific effects of BMAA on central nervous system function, we examined the learning and memory abilities of flies upon BMAA treatment using the aversive phototaxic suppression assay [23,24]. Flies are instinctively phototaxic [32], but quinine, on the other hand, induces avoidance behavior and is frequently used as a negative reinforcer [33,34,35,36]. In the aversive phototaxic suppression assay, flies were individually placed into a T-maze and allowed to choose between a lighted and a darkened chamber. Filter paper is soaked with quinine solution and placed into the lighted chamber such that the quinine provided an aversive association. During the conditioning phase, flies learn to suppress their instinctively phototaxic behavior in order to avoid quinine. Test trials performed immediately post-conditioning (0 hr PC) showed their learning abilities while the test trials performed six hours post-conditioning (6 hr PC) showed their memory abilities. Because this assay requires intact motor ability, we tested the learning and memory ability of flies after three days of feeding with 4 mM BMAA, glutamate or alanine, as this time point marked a climbing pass rate of BMAA-treated flies at around 50% of the control group (data not shown). As shown in Figure 2C, BMAA significantly reduced both learning and memory abilities. Feeding with 4 mM glutamate also significantly reduced the learning ability (0 hr PC), although to a lesser extent compared to BMAA. The memory ability of flies (6 hr PC) fed with glutamate was also reduced, although not as significantly (p = 0.061, one way ANOVA post-hoc Scheffe test). Alanine did not reduce learning and memory abilities by a significant level (p ≥ 0.1 and p ≥ 0.5 respectively, one way ANOVA post-hoc Scheffe test). These results suggest that BMAA induces learning and memory impairment. Glutamate also reduced learning ability, but the effect is much less profound than BMAA.

### 3.2. Dose-dependent Accumulation of BMAA in Flies

**Figure 3:**
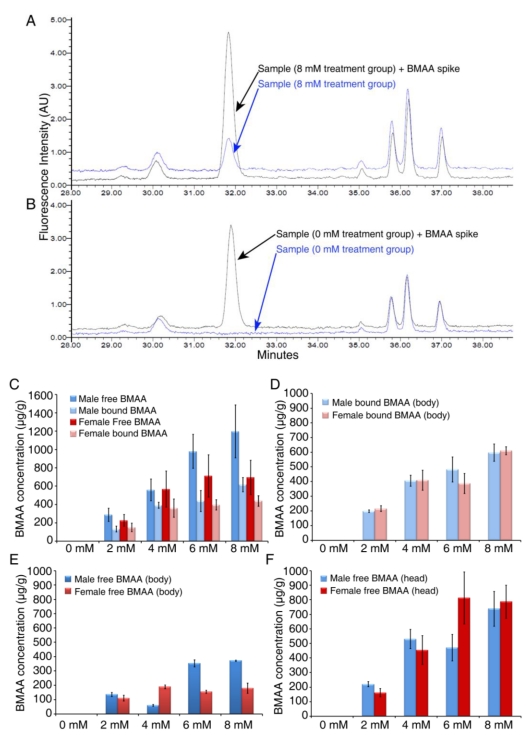
Accumulation of free and protein-bound BMAA in adult flies. Adult flies raised on 0 to 8 mM BMAA for 7 days were collected and extracted for HPLC detection of free and protein-bound BMAA. (A, B) Representative HPLC chromatographic analysis of control sample (A) overlain with a BMAA spike, and treatment (8 mM) sample (B) overlain with a BMAA spike. In whole fly extracts both free and protein-bound BMAA were detected in male and female flies (C). When flies were separated into body and head fractions, it is noteworthy that protein-bound BMAA was found in the body extract (D) but not in the head extract. Free BMAA was detected in both body (E) and head extracts (F). All data were presented as mean ± S.E.M. n = 3.

In order to quantitatively measure the concentration of BMAA in flies after dietary intake and the incorporation of BMAA into fly tissues, we developed an HPLC detection method for *Drosophila* matrix [20], adapted from the previously established method for cyanobacteria [5]. BMAA and the protein amino acids were separated by reverse-phase elution, and the BMAA concentration was determined by detection of the AQC fluorescent tag. The free BMAA fraction is present in the supernatant of tissue extracts, and the protein-bound BMAA fraction is present in the pellet of tissue extracts. Because of the difference in amino acid concentration in the supernatant (free amino acid fraction) and in the pellet (protein-bound fraction), we developed different derivatization methods to achieve optimal detection of free and protein-bound BMAA [20]. Each sample was run with and without a pure BMAA spike to allow the accurate detection of BMAA. As shown in Figure 3A and 3B, overlaying chromatograms from sample runs with and without a BMAA spike revealed BMAA in flies fed with 8 mM BMAA, while no BMAA peak was detected in control flies (0 mM treatment group). The chromatographic peak of BMAA is consistent with previously published results in cycads, flying foxes and ALS/PDC patients [5,8,10,11]. Using this HPLC method we have measured the concentration of free and protein-bound BMAA in flies and found a dose-dependent increase of BMAA concentration in both males and females fed with various concentrations of BMAA for seven days (Figure 3C). Interestingly, we detected higher levels of BMAA in the free fraction than in the bound fraction in both males and females, with 1198 ± 287 µg/g free BMAA and 618 ± 78 µg/g bound BMAA in male flies fed with 8 mM BMAA. The level of free BMAA is lower than that detected in flying foxes (3554 µg/g)—the highest level of free BMAA reported so far—and the level of bound BMAA is similar to that detected in Chamorro people with ALS/PDC (627 µg/g) [4,10]. To examine whether BMAA is incorporated into the brain, we separated the fly heads from the bodies and measured the concentration of BMAA in the heads and bodies separately. *Drosophila* heads consist of head cuticle, eyes, antenna, proboscis, and the brain inside. Since the non‑nervous system tissue including the cuticle and a small amount of muscle cells in the proboscis, is a small part of the whole head, and the majority of the head is nervous system tissue, measuring BMAA concentration in the heads would be a good indication of the incorporation of BMAA into the central nervous system. As shown in Figure 3 we detected both free- and protein-bound BMAA in the body (Figure 3D and 3E). However, we only detected free BMAA in the head (Figure 3F). These results suggest that the *Drosophila* feeding model recapitulates the accumulation of BMAA in the Guam ecosystem and the incorporation of BMAA into the brain. More importantly, the dose‑dependent increase of BMAA concentration in flies is correlated with the severity of alterations in the phenotype. Our observation of preferential brain accumulation therefore might explain the neurodegeneration in the central nervous system of Guam ALS/PDC and other neurodegenerative diseases that might be caused by or related to BMAA exposure [11,37].

### 3.3. Developmental Exposure to BMAA Induces Delayed Locomotor Deficits in Aged Flies

**Figure 4:**
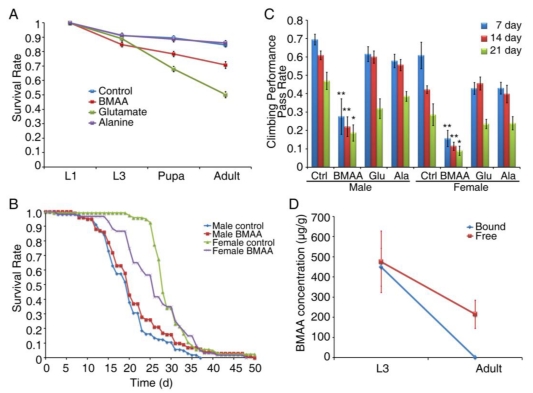
Developmental exposure of BMAA reduced survival rate and caused delayed locomotor deficits in aged flies. Fly embryos were collected and put on sucrose/agar/yeast medium containing 4 mM of BMAA, glutamate or alanine. The number of first instar (L1), third instar (L3) larvae, pupa and adult flies were recorded (A). The eclosed flies were then collected and raised on normal cornmeal/yeast/molasses food. Life span was recorded (B) and climbing performance was tested at 7, 14 and 21 days to examine the progressive degeneration furnished by BMAA (C). The amount of free or protein-bound BMAA was detected in both L3 larvae and adult flies (D). All data were presented as mean ± S.E.M. Significance level was established by t-test. * p ≤ 0.05, ** p ≤ 0.01. n ≥ 10.

Exposure to BMAA during adult stages reduced the life span of *Drosophila* and impaired the *Drosophila* nervous system. To examine the impact of BMAA on development, we collected embryos and raised them on medium containing 4 mM BMAA, alanine or glutamate. The number of surviving flies was recorded at the developmental stages of first instar (L1), third instar (L3), pupa and adult. As shown in Figure 4A, BMAA slightly reduced the survival rate at the pupa and adult stage, while alanine had no effect. Interestingly, glutamate severely reduced the survival rate to 50% at the adult stage (Figure 4A), suggesting that glutamate has more developmental toxicity than BMAA. To examine the effect of early developmental BMAA exposure on adult neurological functions, we collected flies eclosed from larvae fed with BMAA during development and served normal food, and measured their life span and geotactic behavior at 7, 14 and 21 days. Although the life span of adult flies with developmental BMAA exposure was not significantly reduced (Figure 4B), their geotactic behavior was significantly impaired. As shown in Figure 4C, flies with developmental BMAA exposure showed significant reduction in climbing performance at all ages, in contrast to flies exposed to alanine or glutamate, suggesting that developmental exposure to BMAA has a delayed effect on neurological behavior in the adult stages. Next we measured the concentration of BMAA in third instar larvae and in adults eclosed from larvae fed with BMAA. In L3 larvae fed with 4 mM BMAA, we detected 475 ± 153 µg/g of free and 449 ± 94µg/g of protein-bound BMAA (Figure 4D), similar to the levels detected in adult flies fed with 4 mM BMAA (Figure 3C). Interestingly, in 7 day-old adult flies eclosed from BMAA-fed larvae, we detected 215 ± 70 µg/g free BMAA and no protein-bound BMAA (Figure 4D), suggesting that protein-bound BMAA was reduced most likely through proteolysis and free BMAA was partially retained through metamorphosis and aging. Given the significant reduction in climbing performance we observed in 7 day-old flies (Figure 4C), it is possible that either free BMAA may be the primary contributor to the decline in neurological behavior, or that protein-bound BMAA causes a long-lasting and delayed defect, impairing the nervous system after it has vanished.

### 3.4. Developmental Exposure to BMAA Reduces Fertility in Female Flies

**Figure 5:**
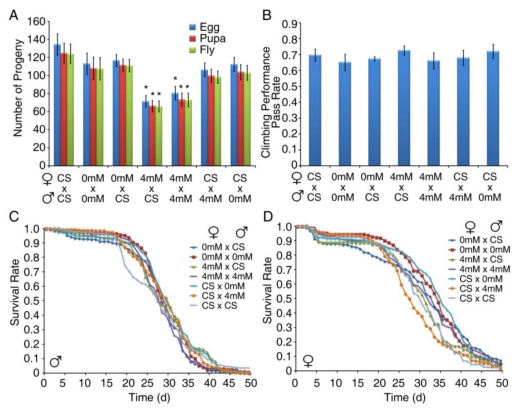
Developmental exposure of BMAA reduced female fertility. Virgin females that eclosed from larvae raised on either normal cornmeal/yeast/molasses food (CS) or sucrose/agar/yeast medium containing 0 or 4 mM BMAA were crossed with 1 day-old males from each group. The crosses were maintained on normal cornmeal/yeast/molasses food. The number of eggs (both fertilized and unfertilized) produced from each cross was recorded daily for ten days, and the number of pupae and adult flies developed from embryos was also recorded (A). Climbing tests were performed on 7 day-old adult progeny from each cross (B). The life span of male (C) and female (D) adult progeny was also recorded. Significance level was determined with one way ANOVA followed by post hoc LSD test. * p ≤ 0.05. n ≥ 7.

To evaluate the impact of BMAA exposure on fertility and the health of progeny, we crossed the virgin females eclosed from larvae raised on either normal food (CS) or sucrose/agar medium containing 0 or 4 mM BMAA, with 1 day-old males from each group, and determined the number of progeny as well as the life span and neurological behavior of adult progeny. As shown in Figure 5A, females that were exposed to 4 mM BMAA produced significantly fewer progeny. However, males that were exposed to BMAA produced the same number of progeny, suggesting that BMAA exposure specifically reduces female fertility. To examine the possible vertical transmission of the toxicity of BMAA from exposed parents to progeny, we raised the progeny on normal food and measured their behavior and life span. The progeny showed no defects in geotactic behavior (Figure 5B) or life span (Figure 5C, male progeny and Figure 5D, female progeny). These results suggest that the toxicity of BMAA was not carried into the progeny, indicating that BMAA did not have lasting effects on germ cells and there was no vertical transmission of the phenotype.

## 4. Discussions

In this report we investigated the comprehensive toxicity of dietary intake of BMAA. We showed that BMAA shortens the life span, impairs locomotor function, and reduces learning and memory abilities in *Drosophila*. We further optimized an HPLC method to reliably detect both free and protein‑bound BMAA in fly tissue extracts. We found that free BMAA is accumulated in the brain and that the severity of the phenotypes is correlated with the concentration of BMAA in flies. We also showed that developmental exposure of BMAA had a limited impact on the survival rate, but significantly reduced fertility in females and induced delayed neurological impairment in aged adults.

### 4.1. Toxic Levels of BMAA in Brain Tissue

The reported detection of BMAA in brain tissue from ALS, PD and AD patients in the United States and Canada [9,10,11,12,13], and the ubiquity of cyanobacteria, support the global presence of BMAA. The potential threat of BMAA on global health necessitates a correlation of the level of BMAA in tissue and its toxicity, and an understanding of the tolerance level of BMAA by an organism. In our studies, the lowest level of BMAA detected in the brain that caused significant behavior defects is 221 ± 17 µg/g in males fed with food containing 2 mM BMAA. Females fed with the same food showed no significant behavior defects and 163 ± 28 µg/g BMAA was detected in the brain (Figure 2 and Figure 3). This observation suggests that BMAA levels of 200 µg/g in the brain might be sufficient to cause neurological defects. Importantly this observation is consistent with the range of BMAA detected in humans. For example, the amount of BMAA detected in postmodern brain tissue from ALS, PD and AD patients in the United States and Canada ranges from 111 to 218 µg/g while the highest level of BMAA detected in non-neurological disease control patients was 45 µg/g [37]. Therefore, accumulation of BMAA in the brain above 100-200 µg/g is likely to increase the susceptibility to neurological diseases, although longer accumulation time in humans may allow even lower concentrations of BMAA to exert detrimental effects.

### 4.2. Progressive Toxicity of BMAA

One feature of Guam ALS/PDC is the prolonged incubation and wash-out periods, indicating that the responsible agent is a chronic neurotoxin [4]. Our results indicate that BMAA exposure has limited impact on development but causes delayed and progressive neurotoxicity. These studies are the first to recapitulate the progressive toxicity of BMAA exposure. Although the mechanisms underlying BMAA-protein interaction remain to be elucidated, it is possible that the ability of BMAA to be incorporated into protein and later released through catabolism extends the time window of its toxicity. It is likely that the protein-bound form of BMAA is less toxic but serves as sink for BMAA [4]. This might also partially explain the limited impact of BMAA exposure during development, as rapid growth and protein synthesis could incorporate BMAA into bound form and reduce the concentration of free BMAA.

### 4.3. BMAA Has Stronger Toxicity than Glutamate

BMAA forms carbamate in the presence of bicarbonate and is only neurotoxic in the presence of bicarbonate [16,38], suggesting that the carbamate is likely to be the toxic agent. Since carbamate BMAA is structurally similar to glutamate, it has been proposed that BMAA induces glutamatergic neurotoxic damage. Results from many studies using cultured mammalian neurons indicate that BMAA produces neurotoxicity by activation of glutamate receptors, and the effects of BMAA can be attenuated by antagonists of both NMDA and AMPA glutamate receptors [15,38,39,40,41,42,43]. In our studies, we observed more severe defects caused by BMAA in adult flies compared to glutamate at the same concentration, while glutamate had a higher impact on survival rate upon developmental exposure. These results suggest that glutamate has more acute toxicity than BMAA, and that BMAA may have other toxic effects in addition to glutamatergic neurotoxic damage. Several possibilities have emerged from recent studies including increased oxidative stress mediated by BMAA inhibition of the cystine/glutamate antiporter Xc- system [44,45] and methylamine, a metabolite of BMAA that could cause oxidative stress and selective depletion of certain amino acids from intact brain cells [46,47]. Further studies are needed to understand the multiple mechanisms by which BMAA produces neurotoxicity.

## 5. Conclusions

Our results suggest that BMAA exposure causes chronic neurotoxicity *in vivo*. Our work also indicates that *Drosophila* is an excellent *in vivo* animal model to study BMAA-induced dementia and motor neuron degeneration. Recently, several *Drosophila* models of ALS have been established by expressing either mutant forms of SOD1 or ALS8/VAB [25,26,27,28]. Our work will serve as a basis for the next phase of research to uncover the mechanisms of BMAA-induced neurodegeneration, to reveal the interaction between environmental and genetic factors, and to identify targets for BMAA that mediate toxicity in motor neurons.
